# Boosting Nanofiltration Membrane Selectivity via Amine‐Polymer Additive Engineering for Efficient Lithium Extraction From Brine

**DOI:** 10.1002/advs.202513172

**Published:** 2025-10-30

**Authors:** Shaofan Duan, Shuai Jiang, Ping Xu, Zhan Li, Pengfei Zhang, Yanyan Liu, Atsushi Matsuoka, Yuqing Lin, Jie Shen, Kecheng Guan, Tomohisa Yoshioka, Hideto Matsuyama

**Affiliations:** ^1^ Research Center for Membrane and Film Technology Kobe University 1‐1 Rokkodaicho, Nada Kobe 657‐8501 Japan; ^2^ Department of Chemical Science and Engineering Kobe University 1‐1 Rokkodaicho, Nada Kobe 657‐8501 Japan; ^3^ School of Materials Science and Engineering Nanyang Environment & Water Research Institute (NEWRI) Nanyang Technological University 50 Nanyang Avenue Singapore; ^4^ Graduate School of Science Technology and Innovation Kobe University 1‐1 Rokkodaicho, Nada Kobe 657‐8501 Japan

**Keywords:** amine‐polymer additive engineering, interfacial polymerization, lithium extraction, nanofiltration membrane

## Abstract

Nanofiltration (NF) membranes used in direct lithium extraction (DLE) from brine offer a more efficient and environmentally friendly alternative to traditional evaporation‐based methods, and are promising for lithium enrichment owing to their ability to selectively reject multivalent cations. However, conventional polyamide (PA) NF membranes prepared using piperazine (PIP) suffer from low Li^+^/Mg^2+^ selectivity due to excessive negative surface charge and a trade‐off between membrane permeance and selectivity. In this study, a scalable and facile strategy to enhance lithium separation performance by incorporating poly(allylamine) (PAA), an amine‐rich polymer, into the aqueous PIP solution as an additive during the interfacial polymerization synthesis of PA is presented. PAA not only introduces additional positive charges into the PA network, improving Mg^2+^ rejection via the Donnan effect, but also alters monomer diffusion, leading to a crumpled PA morphology that contributes to water permeance. The resulting optimum membrane exhibited a high water permeance of 12.1 L m^−2^ h^−1^ bar^−1^, a low LiCl rejection of 12.6 %, and significantly enhanced the rejection of MgCl_2_ from 18.8 % to 94.7 %. Furthermore, a two‐stage NF process using the optimized membrane effectively purified lithium from simulated salt lake brine, highlighting the potential of this strategy.

## Graphical abstract

A diffusion‐controlled interfacial polymerization strategy incorporating poly(allylamine) (PAA) into piperazine (PIP) solution enables precise tuning of amine monomer diffusion and polyamide (PA) network formation with trimesoyl chloride (TMC). The resulting nanofiltration membranes exhibit crumpled morphology, appropriate surface charge, and enhanced Li^+^/Mg^2+^ selectivity, demonstrating an efficient approach for lithium extraction from brines.

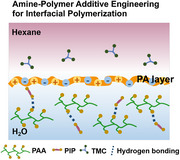

## 1 Introduction

Lithium extracted from natural underground saltwater reservoirs, known as lithium brine, is vital to the global lithium industry, particularly for battery production. However, high concentrations of coexisting ions like Mg^2+^ make lithium extraction significantly more challenging. To meet the need for high‐purity lithium products, efficient Li^+^/Mg^2+^ separation is crucial.^[^ ^1^, ^2^, ^3^, ^4^ ^]^ Compared to traditional methods that rely on cumbersome and time‐consuming evaporation ponds, the emerging direct lithium extraction (DLE) method^[^ ^5^, ^6^ ^]^ that selectively extracts lithium from brines using specialized separation techniques while reinjecting the remaining brine into the reservoir is renowned for its superior efficiency and sustainability.^[^ ^7^, ^8^ ^]^ Membrane‐based separation technology is a promising candidate for the DLE process, benefiting from the small footprint and enhanced separation efficiency.^[^ ^6^ ^]^ Among different membrane processes, nanofiltration (NF) is promising in lithium enrichment by selectively removing multivalent cations while allowing lithium ions to pass through.^[^ ^9^ ^]^ NF membranes primarily operate through size sieving and Donnan exclusion effect, effectively rejecting ions with large hydrated sizes and high valence. This makes them particularly suitable for separating divalent ions such as Ca^2+^ and Mg^2+^ from Li^+^ in salt lake brines.^[^ ^10^, ^11^, ^12^ ^]^ The NF process directly obtains lithium‐rich streams in the membrane permeate, significantly improving the extraction efficiency compared to traditional approach, making it an ideal choice for the DLE process.

NF membranes typically consist of a porous support and an active polyamide (PA) separation layer.^[^ ^13^ ^]^ This PA layer is formed through interfacial polymerization (IP) at the interface between an aqueous solution of amine monomers and an organic solution of trimesoyl chloride (TMC).^[^ ^14^, ^15^, ^16^ ^]^ The resulting properties of this layer are critical for selective Li^+^/Mg^2+^ separation. Therefore, the choice of monomers used in membrane synthesis can directly affect membrane characteristics, significantly influencing separation performance.^[^ ^2^ ^]^ Commercial PA membranes used piperazine (PIP) as the aqueous‐phase monomer to react with TMC.^[^ ^17^, ^18^, ^19^, ^20^, ^21^ ^]^ During the reaction, the presence of water causes excess unreacted acyl chloride groups from TMC to hydrolyze into carboxyl groups, giving the membranes a negative charge. This unfavorably promoting the transport of high‐valence cations like Mg^2+^ and resulting in a low Li^+^/Mg^2+^ selectivity. To reduce this negative charge, strategies such as increasing PIP or lowering TMC concentrations have been employed. However, changing monomer concentrations likely result in overly tight or loose membrane and compromising the permeance or selectivity.^[^ ^22^, ^23^, ^24^, ^25^ ^]^ Beyond PIP monomer, other amine monomers like polyethylenimine, which are rich in amine groups, have been explored to increase the positive charge and improve Mg^2+^ rejection.^[^ ^26^, ^27^ ^]^ Nevertheless, the high density of amine groups tends to form an overly cross‐linked PA network, leading to low permeance and significant Li^+^ rejection.^[^ ^28^, ^29^, ^30^, ^31^ ^]^

Relying on the variation of a single monomer type or its concentration to prepare the NF membranes with desirable Li^+^/Mg^2+^ separation performance is challenging due to the inherent trade‐off effect between permeance and selectivity. Incorporating additives during the IP process provides a promising strategy to control the resulting membrane porosity, structure, or charge.^[^ ^14^, ^32^, ^33^, ^34^, ^35^, ^36^, ^37^, ^38^, ^39^, ^40^, ^41^, ^42^ ^]^ Though improved performance results have been reported, detailed investigation of additive roles in affecting membrane properties and correlation between these properties and Li^+^/Mg^2+^ separation remain underexplored. An ideal additive for lithium‐extraction NF membranes should introduce positively charged groups into the PA network without significantly tightening the network or reducing permeance.^[^ ^32^, ^36^, ^43^ ^]^ Here, we propose using an amine‐rich polymer as an additive to improve the lithium extraction performance of PIP‐based NF membranes. The added amine groups introduce more positive charges, while their excessive cross‐linking with TMC in the PA layer may be mitigated by the competing reaction from PIP. In addition, in aqueous solution, these polymers can interact with PIP to trigger diffusion‐driven instability during IP, resulting in a crumpled PA morphology that improves permeance and offsets potential losses from the increased cross‐linking.^[^ ^44^, ^45^, ^46^, ^47^ ^]^ Overall, higher positive charge density, an optimally tight PA network, and a crumpled structure are expected to largely improve lithium extraction in conventional PIP‐based NF membranes.

**Figure 1:**
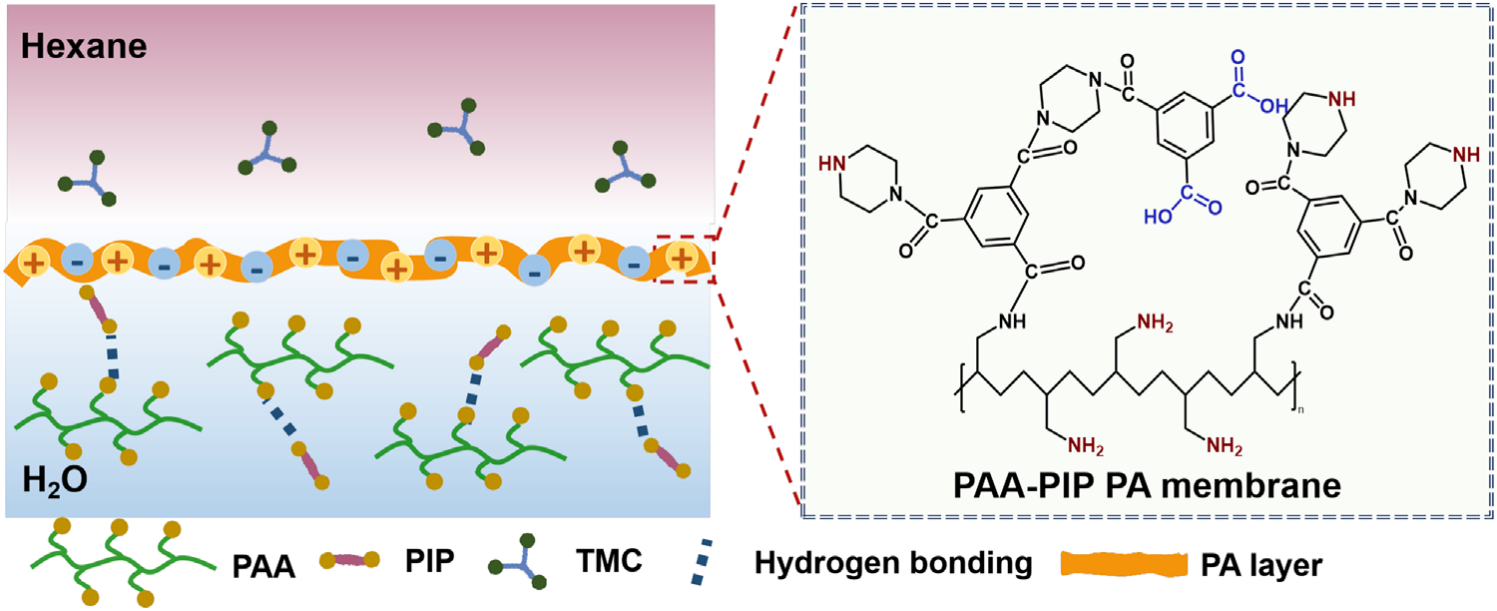
Illustration of IP between an aqueous PAA‐PIP solution and a TMC‐in‐hexane solution, along with the resulting polymer network.

In this work, a fixed concentration of PIP aqueous solution was used with varying concentrations of polyallylamine (PAA) as a polymer additive during the IP process, as shown in **Figure** **1**. Due to its high density of primary amine groups uniformly distributed along a linear polymer backbone, PAA is capable of reacting with TMC without causing significant structural branching in the resulting PA layer.^[^ ^48^, ^49^ ^]^ The aim was to examine how adding PAA influences the diffusion of amine monomers during IP and affects the structure and properties of the resulting PA membrane. PAA can form hydrogen bonds with PIP monomers, which alters PIP diffusion and helps optimize the membrane surface structure. Additionally, incorporating the positively charged PAA will improve the overall positive charge of the NF membrane, potentially enhancing the Li^+^/Mg^2+^ separation selectivity through the enhanced Donnan effect. By fine‐tuning the concentration of PAA, the optimized NF membrane displayed a wrinkled surface structure and a near‐neutral surface charge while achieving high lithium permeance. Moreover, a two‐stage NF process was applied to treat simulated salt lake brine, demonstrating the significant potential of the fabricated membranes for efficient lithium extraction.

## 2 Results and Discussion

### 2.1 Effect of PAA on the PIP Monomer Diffusion

**Figure 2:**
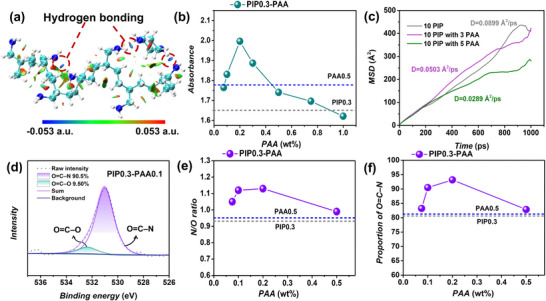
a) Interaction between PIP and PAA monomers by NCI analysis. The blue, green, and white spheres in the map represent nitrogen, carbon, and hydrogen atoms, respectively. The color scale represents the sign(*λ*_2_)*ρ*, ranging from –0.053 a.u. (blue, hydrogen bonding interactions) to +0.053 a.u. (red, steric effect). *ρ* denotes the electron density at the weak interaction critical point, and sign(*λ*_2_) denotes the sign of the second largest eigenvalue *λ*_2_ of the electron density Hessian matrix; The blue regions between adjacent polymer chains highlight hydrogen bonding interactions, as illustrated by the dashed circles. b) Absorbance of diffused amine in hexane with different PAA concentrations over a period of 1 min. The blue and the gray dashed lines indicate the absorbance of bare PIP0.3 and bare PAA0.5 solutions. c) MSD curves for PIP and PIP‐PAA molecules in water. d) XPS O1s spectrum of PIP0.3‐PAA0.1 membrane. e) N/O ratio and f) O=C–N proportion of bare PIP0.3, PIP0.3‐PAA (0.075 wt.%–0.5 wt.%), and bare PAA0.5 membrane, respectively. The blue and the gray dashed lines refer to PAA0.5 and PIP0.3, respectively.

The interaction between PIP monomers and PAA was analyzed via non‐covalent interactions (NCI) analysis, performed using Multiwfn and visualized using Visual Molecular Dynamics (VMD).^[^ ^50^, ^51^, ^52^ ^]^ As shown in **Figure** **2**a, strong hydrogen bonding interactions, including N–H∙∙∙O and O–H∙∙∙N, were observed between PIP and PAA, indicating that PAA can interact with PIP, which may influence the diffusion behavior of PIP during IP process.

To experimentally investigate the impact of PAA on PIP diffusion behavior, interfacial diffusion experiments were conducted between amine solutions (PIP, PAA, and PIP‐PAA) and pure hexane (*n‐*isomer), simulating a practical IP process. The diffused amine in hexane was analyzed using UV–vis spectroscopy (Figure S1a, Supporting Information). Both PIP and PAA in hexane exhibited absorption peaks at ≈195 nm, and changes in absorbance reflected overall amine diffusion. As shown in Figures 2b and S1b (Supporting Information), the absorbance was measured after 1 min diffusion in hexane from a 0.3 wt.% PIP solution (PIP0.3), a 0.5 wt.% PAA solution (PAA0.5), and PIP‐PAA mixture solutions (PIP0.3‐PAAx, where “x” indicates weight concentration of PAA) with PIP at 0.3 wt.% and PAA concentrations ranging from 0.075 to 1.0 wt.%. Compared to PIP0.3, adding small amounts of PAA (0.075 wt.%–0.2 wt.%) in PIP0.3 led to a gradual increase of absorbance with increasing PAA concentration, possibly due to additional diffusion of PAA into hexane alongside PIP. However, as the PAA concentration increased further from 0.2 wt.% to 1.0 wt.%, the absorbance steadily declined, even falling below that of PIP0.3, indicating reduced diffusion of PIP and PAA amine mixtures despite a higher total amine content in the aqueous phase.

To further illustrate the diffusion dynamics, time‐dependent absorbance changes were recorded for representative solution samples of PIP0.3, PAA0.5, and PIP0.3‐PAA0.1 and PIP0.3‐PAA0.5 (Figure S1c, Supporting Information). All normalized absorbance values gradually increased with diffusion time within the first 60 s. Compared with PIP0.3, adding 0.1 wt.% PAA (PIP0.3‐PAA0.1) accelerated the overall diffusion rate, whereas increasing the PAA content to 0.5 wt.% (PIP0.3‐PAA0.5) significantly decreased it. This trend is consistent with the absorbance values measured after 1 min, suggesting that low concentrations of PAA have minimal impact on PIP diffusion; however, at higher concentrations, increased hydrogen bonding between PAA and PIP, along with greater solution viscosity, significantly restrict amine diffusion. Similar additive effects have also been reported in previous studies using comparable approaches.^[^ ^36^, ^53^, ^54^, ^55^ ^]^ The diffusion behavior of pure PAA at different concentrations was also examined (Figure S1d, Supporting Information), showing a decline in absorbance at higher concentrations (0.75 wt.%–1.0 wt.%), confirming the inhibitory effect on its diffusion at high PAA concentrations.

In addition, molecular dynamics (MD) simulations were conducted to verify the inhibitory effect of PAA on the diffusion rate of PIP monomers (Figure 2c). The mean square displacement (MSD) curves revealed that the diffusion rate of PIP in water decreased with the addition of a small amount of PAA (0.0503 Å^2^/ps), compared with bare PIP (0.0899 Å^2^/ps), and a more pronounced reduction (0.0289 Å^2^/ps) was observed at higher PAA contents, indicating the inhibitory effect of PAA on the diffusion of PIP. Overall, the findings suggest that incorporating PAA monomers in the aqueous phase can effectively control amine diffusion, potentially altering the structures of the resulting NF membranes.

### 2.2 Membrane Fabrication, Chemical Structure, and Morphology

PIP0.3‐PAA NF membranes were fabricated on polyethersulfone (PES) support via IP process between corresponding amine monomers and TMC, as illustrated in Figure S2 (Supporting Information). The resulting membranes are denoted as PIP0.3‐PAAx, where x indicates the weight concentration of PAA in the aqueous phase. For comparison, bare PIP and PAA membranes were also prepared using the same procedure and are referred to as PIPx and PAAx, respectively, with x representing the weight concentration of PIP or PAA monomers in the aqueous solution. Fourier‐transform infrared spectroscopy (FTIR) analysis validated the successful fabrication of NF membranes, particularly with the peaks at 1632 and 1400 cm^−1^ associated with the stretching vibrations of the –C═O (amide I band) and C–N bonds, respectively, indicating the effective condensation between amine monomers and acyl chlorides by IP process ^[^ ^48^, ^56^ ^]^ (Figure S3, Supporting Information). X‐ray photoelectron spectroscopy (XPS) analysis (Figure S4, Supporting Information) was further conducted to assess the chemical composition of the synthesized NF membranes (Figure S5 and Table S1, Supporting Information). As the PAA was incrementally introduced in the resulting NF membrane, the chemical composition of the PIP‐PAA membranes progressively transitioned from one resembling PIP membrane to one akin to PAA membrane, particularly when the PAA content reached 0.5 wt.%. Here, primary amine (–NH_2_) groups in PAA, being more reactive toward TMC than the secondary amine (–NH) groups in PIP, preferentially reacted with TMC. Consequently, with increasing the PAA monomer concentration, the membrane composition shifted from being PIP‐dominated to PAA‐dominated. The degree of cross‐linking was evaluated by the N/O ratio and the proportion of N–C═O components from the high‐resolution O 1s spectrum via XPS analysis,^[^ ^57^ ^]^ which increased with PAA incorporation (0.075 wt.%–0.2 wt.%) but slightly decreased for the PIP0.3‐PAA0.5 membrane, approaching that of the PAA membrane (Figure 2d–f; Figure S6, Supporting Information). At low PAA concentrations (0.075 wt.%–0.2 wt.%), the influence of PAA on PIP diffusion was limited, allowing PIP to dominate and form a highly cross‐linked network. In contrast, at higher PAA concentrations, PAA severely restricts the PIP diffusion, thus forming a membrane more closely resembles bare PAA NF membrane with a reduced cross‐linking degree. These results are consistent to the diffusion changes of amine monomers (Figure 2b,c).

**Figure 3:**
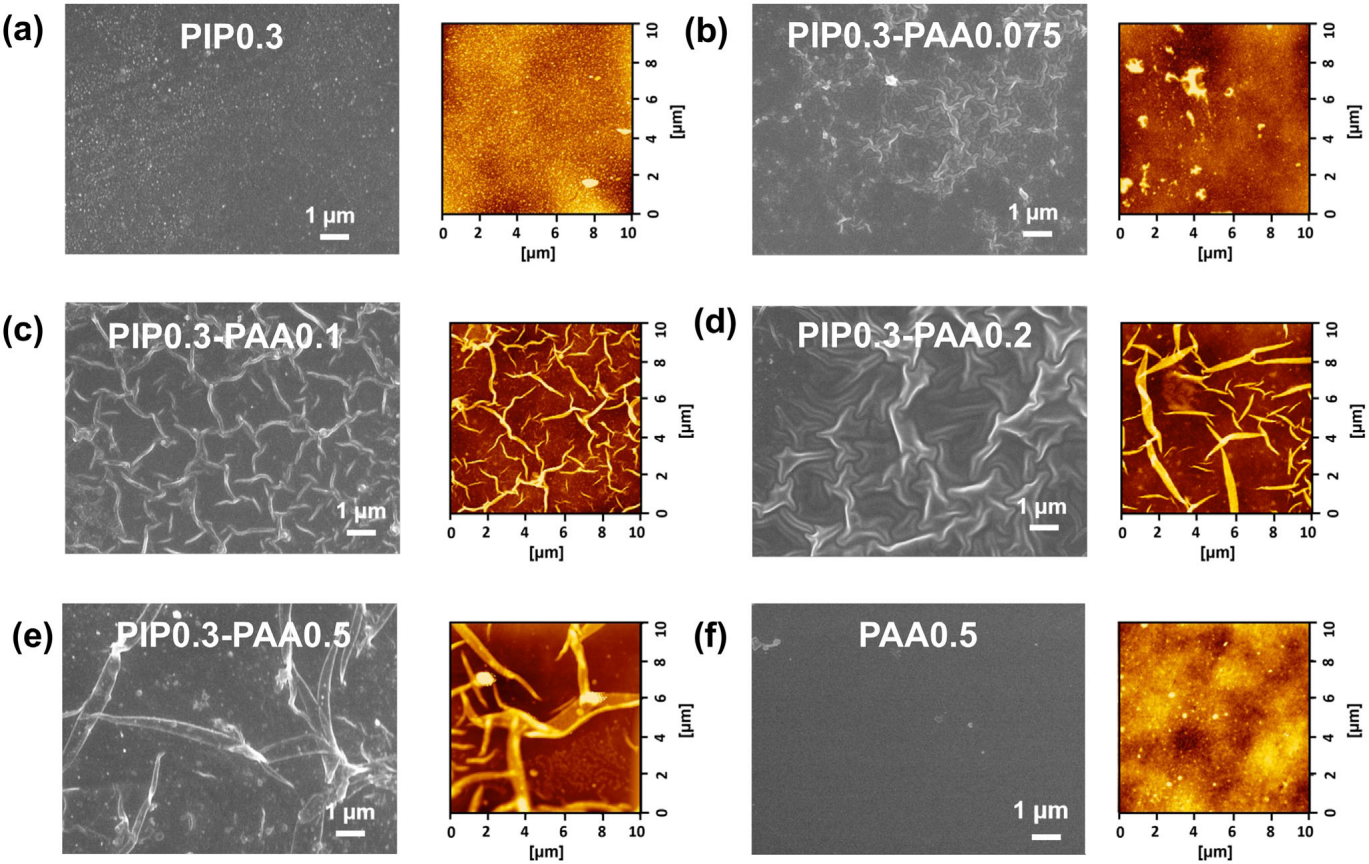
a–f) SEM images and 2D AFM images of the PIP0.3, PIP0.3‐PAA0.075, PIP0.3‐PAA0.1, PIP0.3‐PAA0.2, PIP0.3‐PAA0.5, and PAA0.5 membranes, respectively.

The alteration in the diffusion behavior of PAA and PIP monomers, induced by varying the PAA concentration in the aqueous phase, led to distinct changes in the surface morphology of the resulting PA membranes.^[^ ^58^ ^]^ The membrane surface was characterized by field emission scanning electron microscopy (FESEM) and atomic force microscopy (AFM) to analyze these morphological variations. As shown in **Figure** **3**a, the PIP0.3 membrane shows a typically nodular surface morphology with a relatively low average roughness (7.8 ± 1.7 nm). As the PAA was introduced, nano‐wrinkled structures progressively appeared on the PIP0.3‐PAA membrane surface. The formation of nano‐wrinkled Turing patterns of PIP0.3‐PAA membrane is likely related to the decreased diffusion coefficient caused by the interaction between the PAA and PIP monomers, which leads to diffusion‐driven instability.^[^ ^36^, ^59^ ^]^ The surface roughness of some PIP0.3‐PAA membranes (PIP0.3‐PAA0.1 (12.9 ± 0.7 nm); PIP0.3‐PAA0.2 (12.9 ± 0.9 nm); PIP0.3‐PAA0.5 (20.1 ± 2.2 nm)) were larger than PIP0.3 membranes (7.8 ± 1.7 nm) (Figure 3b–e). In contrast, the PAA0.5 membrane displayed the smooth surface morphology with the lowest surface roughness (2.8 ± 0.4 nm) (Figure 3f). Moreover, the surface morphology of the PIP0.3‐PAA membranes exhibited a gradual transition from a PIP‐like to a PAA‐like appearance, which is consistent with the observed trends in membrane composition and cross‐linking degree (Figure 2e,f). The nano‐wrinkled Turing patterns observed on the PIP0.3‐PAA membranes is expected to increase the effective membrane filtration area, potentially enhancing Li^+^ extraction efficiency. The cross‐sectional morphology of the NF membranes is presented in Figure S7 (Supporting Information). Compared with the PIP0.3 membrane (59.5 nm), the PIP0.3‐PAA membranes exhibited a progressive change in thickness with increasing PAA content (PIP0.3‐PAA0.075 (152 nm); PIP0.3‐PAA0.1 (214 nm); PIP0.3‐PAA0.2 (250 nm); PIP0.3‐PAA0.5 (286 nm)) and eventually showed a thickness comparable to that of the PAA0.5 membrane (295 nm). For PIP‐PAA membranes, the introduction of PAA leads to interactions between the two monomers, resulting in the formation of membranes with thicknesses distinct from those of PIP and PAA membranes. Additionally, the water contact angle (WCA) also proved a noticeable hydrophilicity change (Figure S8, Supporting Information), which is attributed to the altered surface roughness and chemical composition induced by PAA incorporation.

### 2.3 Effect of Pore Size and Surface Charge on Membrane Separation Performance

**Figure 4:**
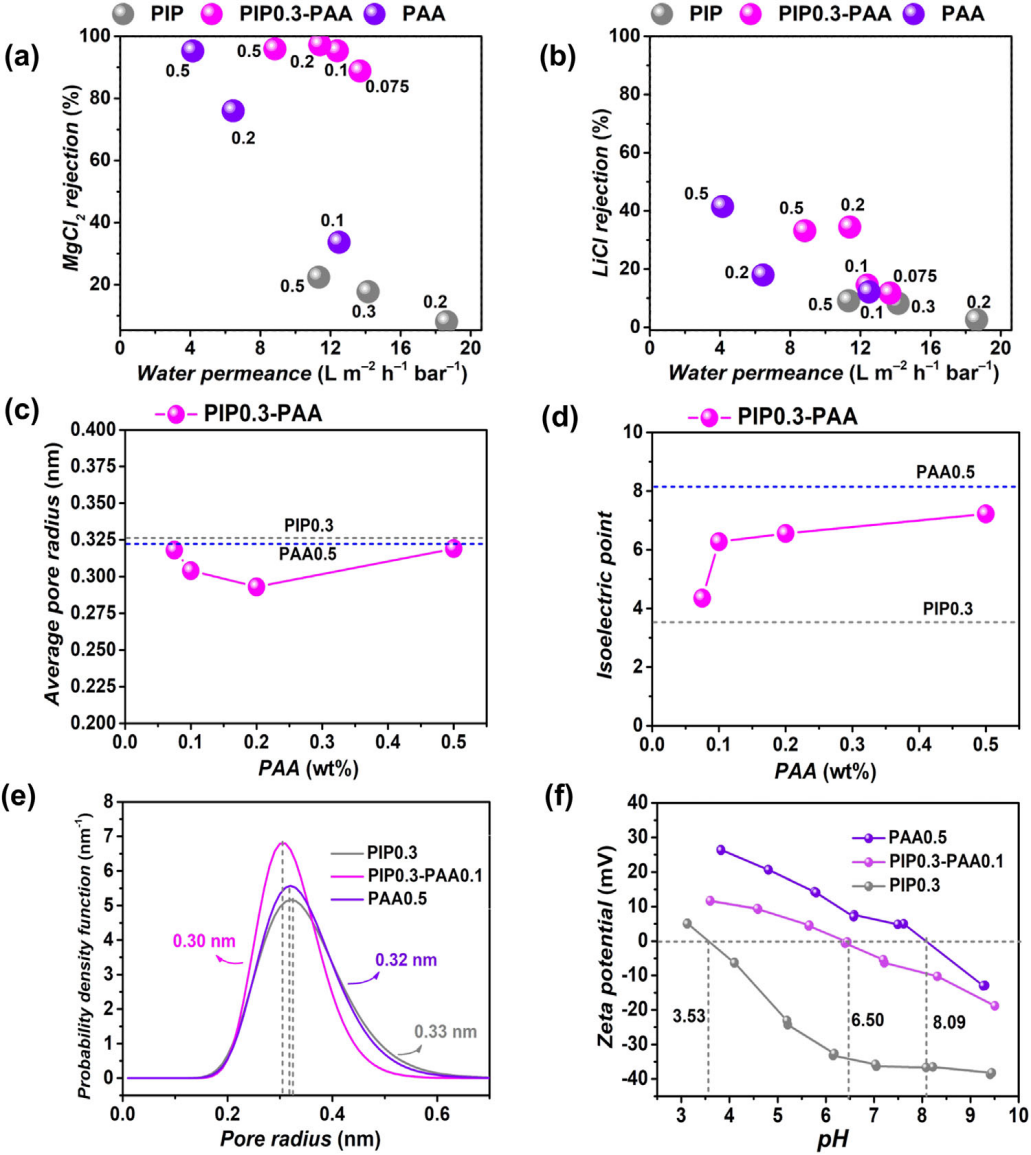
a) MgCl_2_ rejection versus water permeance, b) LiCl rejection versus water permeance plots of prepared PIP (0.2 wt.%–0.5 wt.%), PIP0.3‐PAA (0.075 wt.%–0.5 wt.%), and PAA (0.1 wt.%–0.5 wt.%) membranes. Feed solution: 2000 ppm MgCl_2_ or 2000 ppm LiCl. c) Mean pore radius and d) isoelectric point of PIP0.3, PIP0.3‐PAA (0.075 wt.%–0.5 wt.%), and PAA0.5 membranes. e) Pore size distribution and f) zeta potential of PIP0.3, PIP0.3‐PAA0.1, PAA0.5 membranes, respectively. The data in a and b are shown as mean values, *n* = 3, with error bars excluded from the plots for clarity.

The separation performance of membranes prepared using bare PIP, PAA, and PIP0.3‐PAA was systematically compared (**Figure** **4**a,b). The pure water permeance and rejection of 2000 ppm MgCl_2_ and LiCl single salt solutions for the prepared membranes were evaluated using a crossflow system at a fixed pressure of 5 bar. The bare PIP (0.2 wt.%–0.5 wt.%) membrane exhibited high water permeance but poor MgCl_2_ rejection (Figure 4a,b; Figure S9, Supporting Information). For the PIP0.3‐PAA (0 wt.%–0.5 wt.%) membranes, the water permeance gradually decreased from 14.9 to 9.3 L m^−2^ h^−1^ bar^−1^ but constantly remained higher than that of the bare PAA (0.1 wt.%–0.5 wt.%) membrane. Furthermore, the MgCl_2_ rejection of PIP0.3‐PAA (0 wt.%–0.5 wt.%) membrane increased significantly from 18.8% to 97.2%, while LiCl rejection rose from 7.4% to 36.6% (Figure 4a,b; Figure S10, Supporting Information). It is noteworthy that at low PAA concentrations of 0.075 wt.% and 0.1 wt.%, the LiCl rejection exhibited negligible increases compared to the bare PIP0.3 membrane. Even at a high PAA monomer concentration of 0.5 wt.%, the LiCl rejection (32.5%) of the PIP0.3‐PAA0.5 membrane was still lower than bare PAA0.5 membrane (46.9%). These results suggest that incorporating an appropriate PAA concentration during the preparation of PIP0.3‐PAA membrane can substantially improve MgCl_2_ rejection while maintaining a relatively low LiCl rejection.

To elucidate the reasons for the changes in membrane separation performance, the pore size and surface charge of the bare PIP0.3, PIP0.3‐PAA(0.075 wt.%–0.5 wt.%), and bare PAA0.5 membranes were characterized by the molecular weight cut‐off (MWCO) and surface zeta potential measurements, respectively (Figure 4c–f).^[^ ^60^ ^]^ The PIP0.3 membrane exhibited higher mean pore radius of 0.33 nm and a negative surface charge with an isoelectric point at pH 3.53. Upon the incorporation of PAA (0.075 wt.%–0.5 wt.%), the PA layers with small pore radius were formed and the surface charge of the membranes increased progressively (Figure 4c–f; Figures S11 and S12, Supporting Information). The gradual increase in membrane surface charge can be attributed to the chemical composition of the PIP‐PAA membranes transitioning from resembling PIP membrane to one similar to PAA membrane, as evidenced in amine diffusion experiments, chemical composition analyses (Figure 2), and surface morphology changes (Figure 3). The PIP0.3‐PAA0.1 membrane exhibited a smaller mean pore radius of 0.30 nm and the near‐neutral surface charge with an isoelectric point at pH 6.5. In contrast, the bare PAA0.5 membrane showed a slightly larger mean pore radius of 0.32 nm and a strongly positive surface charge with an isoelectric point at pH 8.14. The increased single salt rejection of PIP0.3‐PAA membrane than PIP membrane was primarily attributed to the combined effects of increased surface charge and the narrowed pore radius. However, the relatively lower LiCl rejection of the PIP0.3‐PAA membranes than PAA0.5 membrane may be due to the relatively neutral membrane surface charge, which weakens electrostatic repulsion against monovalent cations such as Li^+^. In addition, the reduced permeance observed in the PIP0.3‐PAA membrane compared to the PIP membrane can be attributed to two main factors. At low PAA concentrations (0.075 wt.%–0.2 wt.%), PAA provides additional reactive sites that participate in the IP process with PIP and TMC, forming the membrane with small pore size; At higher concentration (0.5 wt.%), the major network was formed by large molecules of PAA, with increased pore size but the numerous entangled long chains rendered more transport resistance (close to the case of bare PAA‐based membrane), further limiting water transport. Nevertheless, the PIP0.3‐PAA membranes exhibited higher water permeance than the bare PAA membranes, likely due to the formation of nano‐wrinkled surface structures that increase the effective filtration area. The optimum PIP0.3‐PAA0.1 membrane that simultaneously exhibits high MgCl_2_ rejection of 94.7%, high water permeance of 12.1 L m^−2^ h^−1^ bar^−1^, and a low LiCl rejection of 12.6%, is ideal for efficient lithium extraction.

To further evaluate the influence of surface charge on separation performance, we compared the different single‐salt separation performance of the optimum PIP0.3‐PAA0.1 membrane with that of the negatively charged PIP0.3 membrane and the positively charged PAA0.5 membrane (Figure S13, Supporting Information). The PIP0.3 membrane exhibited pronounced negatively charged NF membrane behavior, with ion rejection in the following order: Na_2_SO_4_ > MgSO_4_ > MgCl_2_ > NaCl > LiCl. In contrast, the PIP0.3‐PAA0.1 membrane exhibited ion rejection behavior similar to that of PAA0.5 membrane, with the rejection sequence of MgCl_2_ > MgSO_4_ > Na_2_SO_4_ > NaCl > LiCl, consistent with the typical behavior of positively charged NF membranes reported in the literature.^[^ ^61^ ^]^ The relatively lower rejection of Na_2_SO_4_ by the PIP0.3‐PAA0.1 membrane compared to the PIP0.3 membrane was likely attributed to electrostatic interactions from some amine groups on the PIP0.3‐PAA0.1 membrane surface, resulting in a more positively charged structure that weakened the rejection of SO_4_^2−^. Moreover, the decreased Li^+^ rejection compared to the bare PAA0.5 membrane further underscored the dominant role of electrostatic interactions in determining separation performance.

### 2.4 Mixed Li^+^/Mg^2+^ Separation Performance

**Figure 5:**
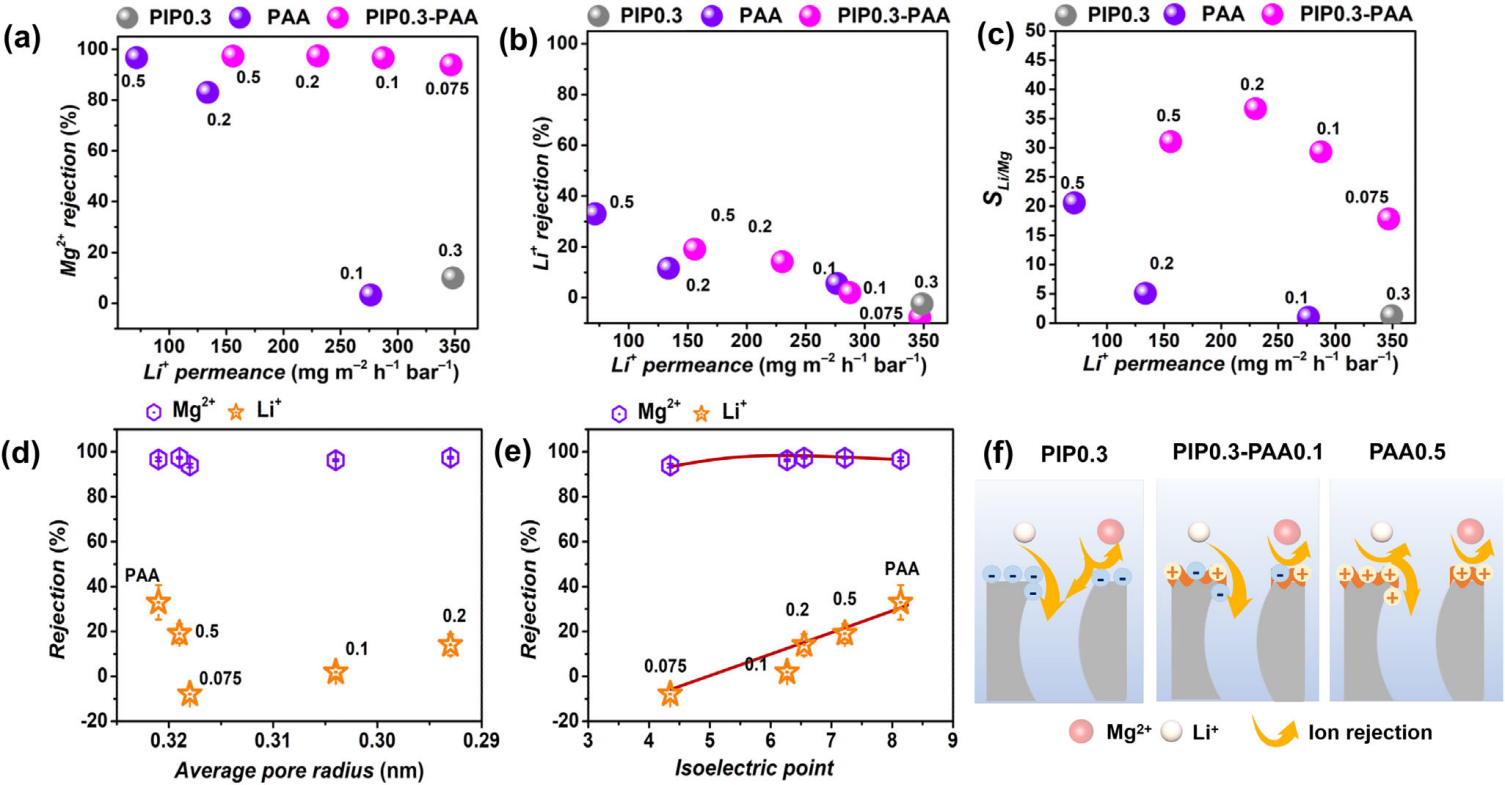
a) Plot of Mg^2+^ rejections versus Li^+^ permeance, b) plot of Li^+^ rejections versus Li^+^ permeance, and c) plot of *S_Li/Mg_* versus Li^+^ permeance of prepared PIP0.3, PIP0.3‐PAA (0.075 wt.%–0.5 wt.%), and PAA (0.1 wt.%–0.5 wt.%) membranes, respectively. Feed solution: 2000 ppm MgCl_2_ and LiCl mixture solution (Mg^2+^/Li^+^ mass ratio: 20). The correlation of ion rejection of PA membrane with d) average pore size and e) isoelectric point. f) Li^+^/Mg^2+^ separation mechanism of PIP0.3, PIP0.3‐PAA0.1 and PAA0.5 membrane. The data in a, b, and c are shown as mean values, *n* = 3, with error bars excluded from the plots for clarity; whereas data in d and e are shown as mean ± SD, *n* = 3.

To further highlight the advantages of the PIP0.3‐PAA membrane over bare PAA and PIP0.3 membranes, we investigated the Li^+^/Mg^2+^ separation performance of the membranes using a mixed salt solution containing LiCl and MgCl_2_ with a Mg^2+^/Li^+^ mass ratio of 20. The experiments were conducted on a crossflow testing equipment under an applied pressure of 5 bar. These membranes showed a similar performance trend to that observed in single‐salt systems (**Figure** **5**a,b; Figure S14, Supporting Information). Notably, a negative rejection phenomenon was observed for Li^+^, indicating that increased permeation of Li^+^ arises from the strong rejection of Mg^2+^, which suppresses Mg^2+^ diffusion and thereby drives Li^+^ transport through an ion‑coupled diffusion mechanism to maintain electroneutrality with the permeated Cl^−^ in the permeate.^[^ ^62^, ^63^, ^64^ ^]^ The calculated Li^+^ permeance, separation factor (*S_Li/Mg_*) values, and lithium recovery (*LiR*) were used to assess the separation performance of Li^+^/Mg^2+^ as well as lithium recovery efficiency (Figure 5c; Figure S15, Supporting Information). The PIP0.3 membrane exhibited high Li^+^ permeance and *LiR* but a very low *S_Li/Mg_*, while bare PAA membranes showed relatively high *S_Li/Mg_* but low Li^+^ permeance and *LiR*. In contrast, PIP0.3‐PAA membranes are much favorable in these separation properties. Particularly, the PIP0.3‐PAA0.1 membrane showed optimal combinations of *S_Li/Mg_*, Li^+^ permeance, and *LiR* than bare PAA0.5 membrane, which is more advantageous for efficient lithium recovery.

In addition, we correlated the ion rejection of PA membrane with mean pore radius and isoelectric point to further reveal their roles in ion rejection (Figure 5d,e). The ion rejection showed good dependence with the isoelectric point rather than the mean pore size, emphasizing the importance of surface charge regulation in Li^+^/Mg^2+^ separation.^[^ ^58^ ^]^ The proposed Li^+^/Mg^2+^ separation mechanism for the PIP0.3, PIP0.3‐PAA0.1, and PAA0.5 membranes are illustrated in Figure 5f. For the negatively charged PIP0.3 membrane, it exhibits high attraction for the positively charged Mg^2+^, resulting in low rejection due to the opposite charges. While for PAA0.5 membrane, it has higher positive charge on its surface, leading to high rejection of Mg^2+^ but also certain rejection of Li^+^. The PIP0.3‐PAA0.1 membrane exhibits a nearly neutral surface charge and balances electrostatic interactions, allowing the facile passage of monovalent ions like Li^+^ while effectively reject Mg^2+^ based on the pore size.^[^ ^65^, ^66^ ^]^ Overall, within a certain pore size range, regulating the membrane surface charge plays a crucial role in adjusting the membrane separation performance. This work, by precisely adjusting the surface charge of the PIP‐based membrane, high lithium permeance and excellent Li^+^/Mg^2+^ selectivity can be simultaneously achieved, offering a promising strategy for efficient lithium extraction.

### 2.5 Practical Feasibility of Separating Li^+^/Mg^2+^

**Figure 6:**
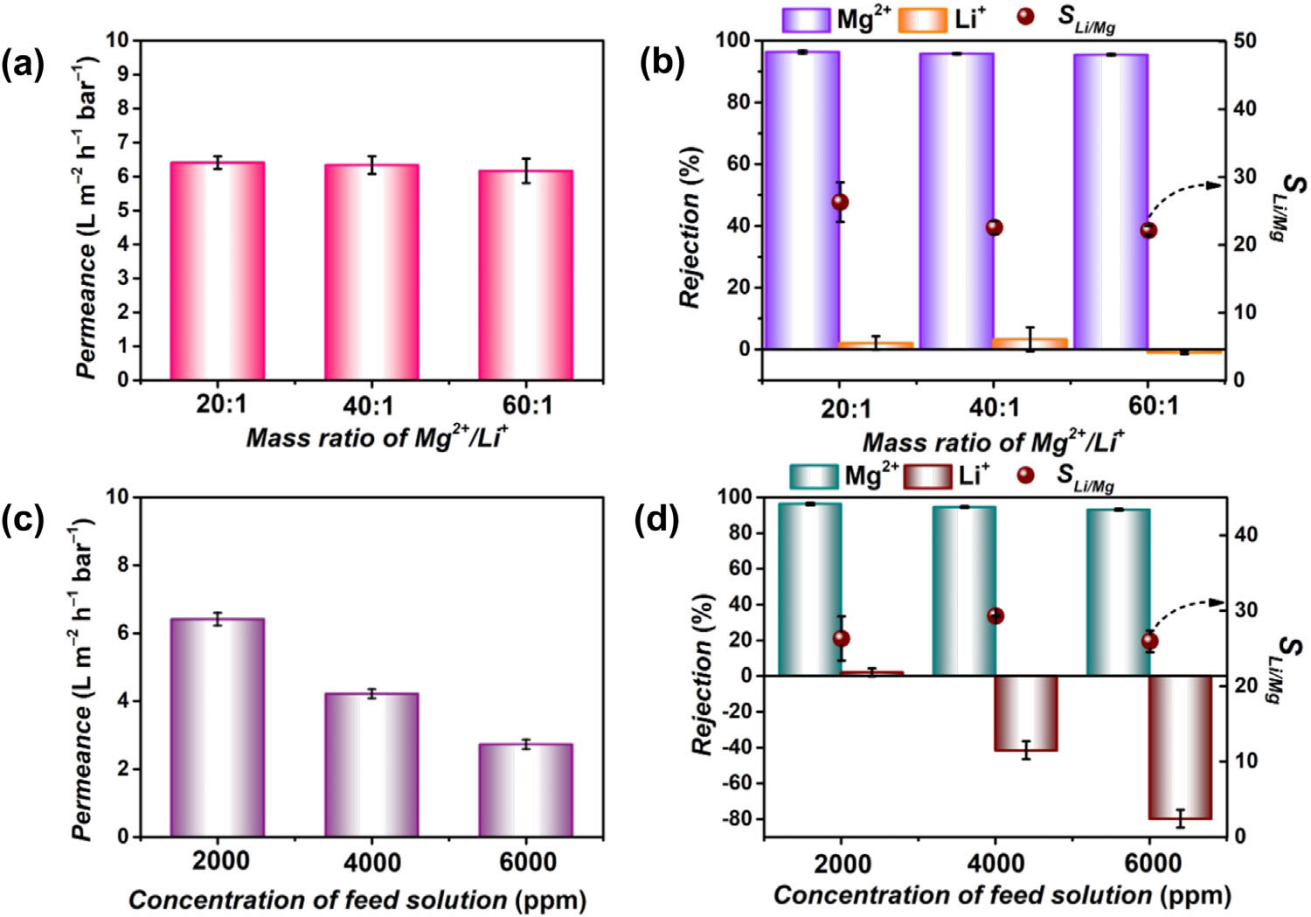
a) Permeance, b) ion rejection, and *S_Li/Mg_* of PIP0.3‐PAA0.1 membrane by filtrating MgCl_2_ and LiCl mixture solution with varying Mg^2+^/Li^+^ mass ratios (20, 40, and 60) at a total salt concentration of 2000 ppm. c) Permeance and d) ion rejection, and *S_Li/Mg_* of PIP0.3‐PAA0.1 membrane by filtrating MgCl_2_ and LiCl mixture solution with different total salt concentrations (2000, 4000, and 6000 ppm) at a Mg^2+^/Li^+^ mass ratio of 20. The data in a‐d are shown as mean ± SD, *n* = 3.

It has been reported that salt lakes from different regions contain high concentrations Mg^2+^ and varied Mg^2+^/Li^+^ mass ratios, increasing the difficulty of efficient lithium extraction.^[^ ^27^ ^]^ Hence, we prepared simulated brines with different concentrations and Mg^2+^/Li^+^ mass ratios as feed solutions to evaluate the separation performance using the optimum PIP0.3‐PAA0.1 membrane. As shown in **Figure** **6**a,b, increasing the Mg^2+^/Li^+^ mass ratio had little impact on the permeance and Li^+^/Mg^2+^ separation. However, membrane permeance significantly decreased with higher total concentration of the feed solution (Figure 6c), due to the variation in osmotic pressure, consistent with other studies.^[^ ^63^ ^]^ Although Mg^2+^ rejection slightly decreased in this case, Li^+^ rejection dropped more to a significantly negative level, resulting in nearly constant Li^+^/Mg^2+^ selectivity with minor fluctuations (Figure 6d).

The durability of the PIP0.3‐PAA0.1 membrane was assessed through a 7‐day continuous separation operation. As shown in Figure S16 (Supporting Information), the permeance and ion rejection of the membrane remained constant during the test. In addition, an inorganic fouling/scaling test (Figure S17) using feed solution containing 300 mg L^−1^ Na_2_SiO_3_, 500 mg L^−1^ CaSO_4_, 500 mg L^−1^ MgCl_2_, and 500 mg L^−1^ LiCl was conducted to evaluate the membrane resistance to typical inorganic contaminants commonly present in brines. Ca^2+^, SO_4_^2−^, and SiO_3_^2−^ can form insoluble salts and silicate precipitates that deposit on the membrane surface, leading to permeance decline.^[^ ^67^ ^]^ The normalized permeance of both PIP0.3 and PIP0.3‐PAA0.1 membranes showed a declining trend during the test. Although the pure water permeance exhibited only a slight decrease after water rinsing, clear particle deposition observed in SEM images for both membranes after the inorganic fouling/scaling tests (Figure S18, Supporting Information), indicating the importance of future surface modification strategies to enhance scaling resistance of the membrane.

A separation performance comparison of the membrane prepared in this work with those reported in other literature is presented in Figure S19 and Table S2 (Supporting Information). The incorporation of PAA during the IP process played a beneficial regulatory role, resulting in the membrane showing distinct advantages in lithium permeance and Li^+^/Mg^2+^ separation selectivity, highlighting its significant potential in lithium recovery applications.

**Figure 7:**
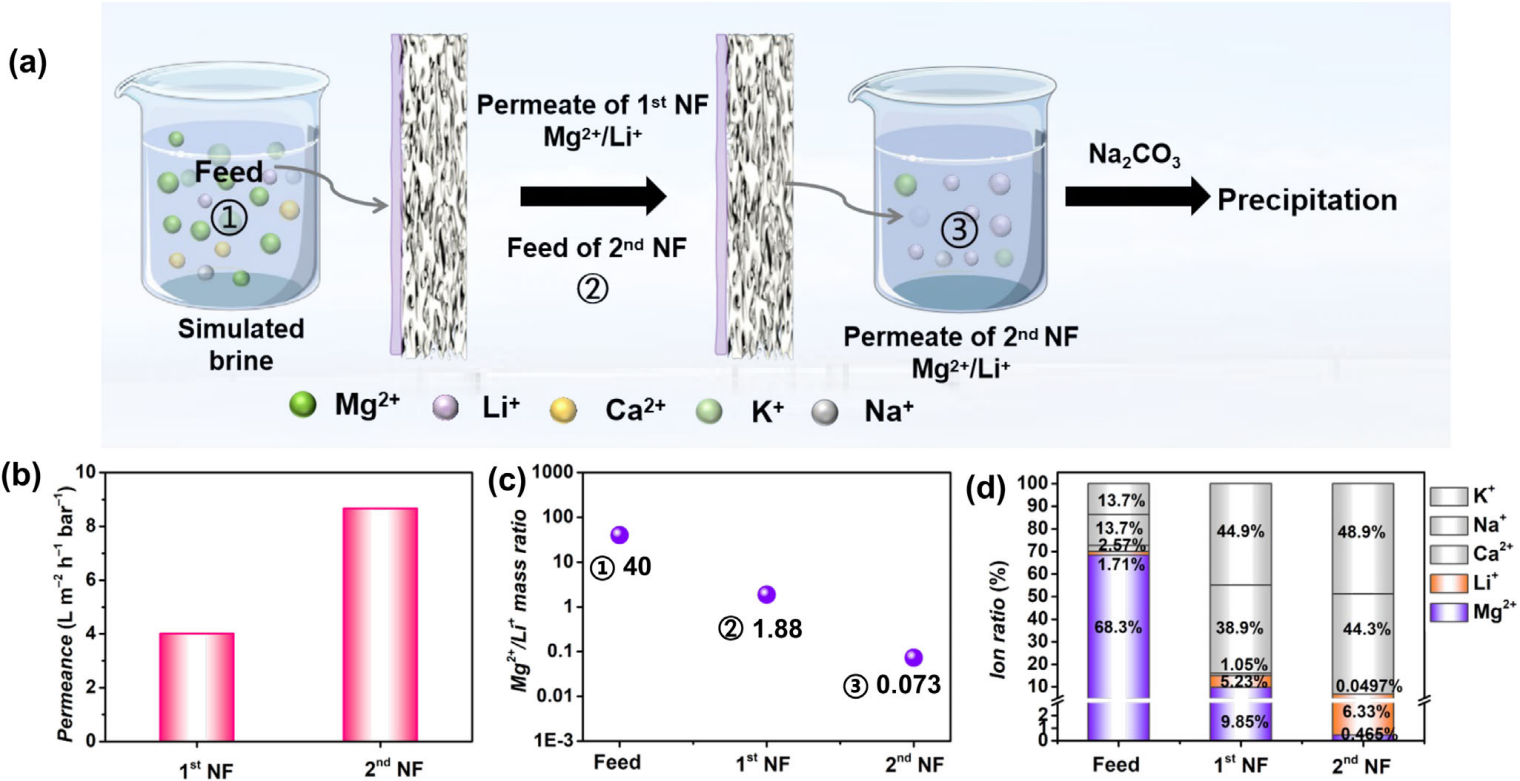
a) Two‐stage NF process for treating the simulated salt lake brine. b) Permeance and c) mass ratio of Mg^2+^/Li^+^ of feed solution, 1st, and 2nd NF process. d) Ion mass ratio in the feed and permeate solutions among 1st and 2nd NF process.

Additionally, we prepared a multi‐ion feed solution simulating the Taijinar salt lake brine to assess the practical lithium extraction performance of the PIP0.3‐PAA0.1 membrane in a two‐stage NF process. The simulated salt lake brine had a Mg^2+^/Li^+^ mass ratio of 40, while the experimental test was conducted under a pressure of 5 bar. The permeate from the 1st NF process served as the feed solution for the 2nd NF process, with the specific procedure shown in **Figure** **7**a. The permeance significantly increased after the two‐stage NF process (Figure 7b), primarily due to the reduced concentration. After the two‐stage NF process, the Mg^2+^/Li^+^ mass ratio decreased substantially from 40 in the original feed solution to 0.073 (Figure 7c). Figure 7d also illustrates the different ion ratios in the various solutions during the two‐stage NF process. The Mg^2+^ concentration dropped from initial 1200 to 2.15 ppm in the final solution, while its proportion in the total cation content dropped from 68.3% to 0.5%. Notably, the Li^+^ ratio increased compared to the feed solution, indicating selective enrichment of lithium. The significant reduction in the Mg^2+^/Li^+^ mass ratio coupled with the effective enrichment of lithium ions further demonstrates the potential of the PIP‐PAA membrane for lithium extraction in practical applications. Absolute Li^+^ concentrations at each stage are summarized in Table S3 (Supporting Information). The Li^+^ concentration in the 2nd‐stage permeate (29.3 ppm) was similar to that of the feed solution (30.0 ppm). Nevertheless, a subsequent concentration step is necessary for effective recovering by precipitation.^[^ ^10^ ^]^

Practically, specific Li^+^ recovery from the membrane process is strongly dependent on the water recovery of original feed (Figure S20, Supporting Information). Besides, other monovalent cations (e.g., Na^+^, K^+^) also permeate with Li^+^, and their concentrations may need to be reduced in certain cases that may affect Li_2_CO_3_ precipitation.

## 3 Conclusion

Overall, we successfully designed and fabricated PA membranes with nano‐wrinkled structure and enhanced positive charge for lithium extraction by incorporating amine‐rich PAA to the aqueous PIP monomer solution during the IP process. The addition of PAA effectively reduce the diffusion rate of the PIP monomers, while the abundant amine groups in the PAA enhanced the positive charge of membrane. The results demonstrated the effectiveness of the amine polymer in modulating the nanostructure and surface charge of the membrane. We systematically investigated the impact of different concentrations of PAA on the membrane formation process and its Li^+^/Mg^2+^ separation performance, revealing the significant role of charge regulation in membrane separation performance. The optimum PIP0.3‐PAA0.1 membrane exhibited high pure water permeance of 12.1 L m^−2^ h^−1^ bar^−1^, with a high Li^+^/Mg^2+^ selectivity of 29.7. Furthermore, a two‐stage NF process using the optimum membrane significantly reduced the Mg^2+^/Li^+^ mass ratio in simulated salt lake brine from 40 to 0.073, further demonstrating the great potential for selective lithium recovery. This work highlights the substantial advantages of incorporating positively charged additives during the IP process to optimize membrane surface charge and morphology, offering an effective and scalable strategy for preparing NF membranes for efficient lithium extraction.

## 4 Experimental Section

### Chemicals and Materials

PES (Synder flat sheet membrane UF membrane; MWCO: 20,000 Da) purchased from Sterlitech Corporation (USA) was used as the substrate for membrane fabrication. PAA (15% aqueous solution; MW: 15 000 g mol^−1^) was supplied by Polysciences, Inc (PA, USA). TMC was obtained from Tokyo Chemical Industry Co., Ltd. (Tokyo, Japan). PIP and hexane were obtained from FUJIFILM Wako Pure Chemical Co., Tokyo, Japan. Deionized water was prepared using a Milli‐Q Integral 3 system supplied by Millipore S.A.S., Molsheim, France.

MgCl_2_·6H_2_O, CaCl_2_, MgSO_4_, Na_2_SO_4_, NaCl, KCl, LiCl, CaSO_4_·2H_2_O, and Na_2_SiO_3_·9H_2_O, with different salt compositions and proportions, as supplied by FUJIFILM Wako Pure Chemical Co., Tokyo, Japan, were used for single salt test, the preparation of simulated salt lake brines, and inorganic fouling/scaling test. Sucrose and D‐(+)‐glucose (180 Da) supplied by Tokyo Chemical Industry Co., Ltd. Tokyo, Japan as well as Glycerol and D‐(+)‐raffinose purchased from FUJIFILM Wako Pure Chemical Co., Tokyo, Japan, were used for MWCO measurements.

### NF Membrane Preparation Method

The NF membranes were prepared on PES substrate by IP process, as detailed in Figure S2 (Supporting Information). To prepare PIP‐based PA membranes with PAA additive, the aqueous PIP solution at 0.3 wt % was fixed and sequentially added varying concentration of PAA (0.075 wt.%, 0.1 wt.%, 0.2 wt.%, and 0.5 wt.%). The prepared solution was poured onto the PES membrane for 2 min, and then the excess aqueous solution was removed from the surface of the membrane. Next, a 0.1 wt.% TMC hexane solution was slowly poured over the membrane for 1 min to initiate cross‐linking, followed by heating in an oven at 70 °C for 3 min. The resulting membranes, denoted as PIP0.3‐PAAx (x represents the weight concentrations of PAA monomers), were stored in deionized water at 4 °C for future use. The bare PIP (0.2 wt.%, 0.3 wt.%, and 0.5 wt.%) and PAA (0.1 wt.%, 0.2 wt.%, and 0.5 wt.%) membranes were also prepared and compared with those PIP0.3‐PAA membranes, using a consistent preparation method throughout. The preparation conditions of different NF membrane types were shown in Table S4 (Supporting Information).

### Characterization

An UV‐vis spectrometer (V‐650KE, JASCO Company, Tokyo, Japan) was used to investigate the diffusion behavior of the PAA, PIP and PIP‐PAA monomers from the aqueous solution to the hexane. AFM (SPI3800 N; JEOL, Ltd, Tokyo, Japan) was employed to assess the surface roughness of the NF membranes. FESEM (JSF‐7500F, Hitachi Ltd, Tokyo, Japan) was utilized to examine the impact of varying PAA concentrations on the surface microstructures of the membranes. FTIR (ALPHA, Bruker, Billerica, MA, USA) and XPS (JPS‐9010 MC, JEOL, Ltd., Tokyo, Japan) were used to analyze the chemical composition and structure of membranes. WCA values, which were used to compare the hydrophilicity of different membranes, were obtained using a contact angle goniometer (Drop Master 300; Kyowa Interface Science Co. Ltd., Tokyo, Japan). Each measurement repeated at least three times. The zeta potential of the membranes was measured and characterized using an electrokinetic analyzer (Anton Paar SurPASSTM 3, Graz, Austria) to observe membrane surface charge. Data errors in this study were quantified using standard deviation.

### NF Membrane Separation Performance Evaluation

The NF membrane separation performance test was performed using crossflow testing equipment with an effective filtration area of 7.06 cm^2^. For each test, a fixed pressure of 5 bar was applied to effectively overcome the osmotic pressure of different feed salt solutions, with a pre‐pressurization time at least 60 min for the NF membrane. Additionally, data was collected once the permeate solution mass and conductivity remained stable. The water permeance and permeance of mixed solution (*P*, L m^−2^ h^−1^ bar^−1^) of the NF membrane was calculated using the following formula:^[^ ^68^ ^]^

(1) $$P=\frac{V}{S\times \Delta t\times P}$$

where *V* (L) denotes the volume of permeate collected over a specified filtration time (*∆t*, h), *P* is the constant test pressure (bar), and *S* (m^2^) represents the effective filtration area of the membrane. The rejection *R* (%) of the NF membrane for different salts or ions was determined using the following formula:

(2) $$R=\left(1-\frac{Cp}{Cf}\right)\times 100\%$$

where *C_p_* and *C_f_* are salt or ion concentrations (ppm) in the permeate and feed, respectively. Single salt solution test was performed using 2000 ppm concentration of different salts. Mixed MgCl_2_/LiCl solutions with varying concentrations (2000, 4000, and 6000 ppm) at a fixed Mg^2+^/Li^+^ mass ratios of 20 as well as varying Mg^2+^/Li^+^ mass ratios (20, 40, and 60) at a fixed total concentration of 2000 ppm were used to evaluate the Li^+^/Mg^2+^ mixture separation performance of the NF membranes. The separation factor (*S_Li/Mg_*) was calculated using Equation (3).

(3) $$S_{Li/Mg}=\frac{Cp,Li/Cp,Mg}{Cf,Li/Cf,Mg}$$

where *C_f, Mg_* and *C_p, Mg_* is the Mg^2+^ concentrations (ppm) at the feed and permeate solution, respectively. *C_f, Li_* and *C_p, Li_* is the Li^+^ concentrations (ppm) at the feed and permeate solution, respectively, which were measured using an inductively coupled plasma optical emission spectrometer (ICP‐OES; Shimadzu, Japan).

Additionally, the lithium recovery (*LiR*) is determined using Equation (4):^[^ ^69^ ^]^

(4) $$LiR=WR\left(1-R_{Li}\right)$$

*WR* is water recovery and *R_Li_* is the Li^+^ rejection of the NF membrane. In this study, a water recovery of 50% and water‐recovery independent rejection performance for the calculation was assumed.

A simulated Taijinar salt lake solution was prepared using 183 mg L^−1^ LiCl, 4700 mg L^−1^ MgCl_2_, 609 mg L^−1^ NaCl, 457 mg L^−1^ KCl, and 124 mg L^−1^ CaCl_2_, and was used as the feed solution for the two‐stage NF process under a pressure of 5 bar to explore the potential of the PIP0.3‐PAA0.1 membranes for lithium extraction.^[^ ^65^, ^70^ ^]^ In this process, the feed solution for the secondary NF process consists of the permeate obtained from the primary NF process.

### MWCO Test

1000 ppm aqueous solutions of glycerol (92 Da), sucrose (342 Da), (+)‐glucose (180 Da), and D‐(+)‐raffinose (504 Da) were used for membrane rejection tests to measure the MWCO values and obtain pore size distribution of the prepared membranes. The test was conducted at a constant pressure of 5 bar and the solute concentrations in both the feed and permeate solutions were measured using a total organic analyzer (TOC‐VCSH, Shimadzu, Japan). The MWCO values were defined as the molecular weight at which 90% of the sugar solutes was rejected by the membrane. In addition, the pore size distribution of the membranes was determined using the following formula:^[^ ^30^ ^]^

(5) $$\frac{dR(d_{p})}{dd_{p}}=\frac{1}{\sqrt{2\pi }dp\ln\sigma p}\exp\left[-\frac{{\left(\ln dp-\ln\mu p\right)}^{2}}{2{\left(\ln\sigma p\right)}^{2}}\right]$$

where *d_p_* is the Stokes radius of the sugar molecules. *µ_p_* refers to the mean pore diameter, calculated at the sugar solute rejection of 50%. *σ_p_* denotes for the geometric standard deviation, determined by the ratio of *d_p_* at a sugar rejection of 84.13% to that at 50%.

### Computational Simulations

To investigate the non‐covalent interactions between PIP and PAA, NCI analysis was performed. The molecular models of PIP and PAA were first constructed and combined to form the PIP‐PAA system, followed by structural optimization to reach the minimum energy configuration. The Multiwfn package was then employed to conduct the NCI analysis, and the results were visualized using the VMD software. The modeling protocol was consistent with that described below for the MD simulations.^[^ ^50^, ^51^, ^52^ ^]^

To investigate the effect of PAA incorporation on PIP diffusion, MD simulation was performed. Specifically, a PAA oligomer consisting of seven repeating units was constructed as a representative model. Three simulation systems were then generated using the Amorphous Cell module: i) a pure PIP–water system containing 10 PIP molecules and 2000 water molecules; ii) a mixed PIP/PAA solution with a lower PAA concentration (10 PIP molecules, 3 PAA molecules, and 2000 water molecules); and iii) a mixed PIP/PAA solution with a higher PAA concentration (10 PIP molecules, 5 PAA molecules, and 2000 water molecules). All systems were subjected to geometry optimization to minimize energy, followed by a 1000 ps NVT ensemble simulation using the Forcite module. The MSD and diffusion coefficient (*D*) of PIP and PAA in each system were subsequently analyzed and calculated using the following equation:

(6) $$MSD\left(t\right)=\frac{1}{N}\sum_{i=1}^{N}\langle {\left[ri(t)-ri(0)\right]}^{2}\rangle$$

(7) $$D=\frac{1}{6t}MSD\left(t\right)$$

It should be noted that the concentrations of amine monomers in these simulations were intentionally set higher than those in actual experimental conditions to enhance sampling efficiency and reduce computational cost, which was a widely adopted practice in similar studies.^[^ ^71^, ^72^ ^]^

## Conflict of Interest

The authors declare no conflict of interest.

## Supporting information



Supporting Information

## Data Availability

The data that support the findings of this study are available from the corresponding author upon reasonable request.
