# Supplementary material for: Boosting Nanofiltration Membrane Selectivity via Amine‐Polymer Additive Engineering for Efficient Lithium Extraction From Brine
Source: Adv Sci (Weinh). 2025 Oct 30;13(3):e13172. doi: 10.1002/advs.202513172 (PMC12806463; doi:10.1002/advs.202513172)
Supplement: Supplementary file 1 — Supporting Information [file ADVS-13-e13172-s001.pdf]

Supporting Information

Boosting Nanofiltration Membrane Selectivity via Amine-Polymer Additive Engineering for Efficient Lithium Extraction from Brine

Shaofan Duan[a,b], Shuai Jiang[a,b], Ping Xu[a], Zhan Li[a], Pengfei Zhang[a], Yanyan Liu[a], Atsushi Matsuoka[a,b], Yuqing Lin[a], Jie Shen[c], Kecheng Guan[a,*], Tomohisa Yoshioka[a,d], Hideto Matsuyama[a,b,*]

[a] *Research Center for Membrane and Film Technology, Kobe University, 1-1 Rokkodaicho, Nada, Kobe, 657-8501, Japan*
[b] *Department of Chemical Science and Engineering, Kobe University, 1-1 Rokkodaicho, Nada, Kobe, 657-8501, Japan*
[c] *School of Materials Science and Engineering, Nanyang Environment & Water Research Institute (NEWRI), Nanyang Technological University, 50 Nanyang Avenue, Singapore*
[d] *Graduate School of Science, Technology and Innovation, Kobe University, 1-1 Rokkodaicho, Nada, Kobe, 657-8501, Japan*

Corresponding authors:

Kecheng Guan, e-mail: guan@people.kobe-u.ac.jp

Hideto Matsuyama, e-mail: matuyama@kobe-u.ac.jp

Diffusion experiment of PIP, PAA and PIP-PAA monomer from aqueous phase to organic phase

The diffusion behavior of the PIP, PAA or PIP-PAA monomers from an aqueous solution into the hexane phase was analyzed using a UV-vis absorption spectrometer (V-650KE, JASCO Company, Japan). In the experiment, 25 mL of an aqueous solution containing either pure PIP0.3, pure PAA (0.1–1.0 wt%), or PIP0.3-PAA (0.075–1.0 wt%) mixture at varying concentrations was placed at the surface of pristine PES support for 2 minutes. Excess solution was then removed with an air knife. Subsequently, 20 mL of pure hexane was gently added on top of the support and allowed to interact for 1 minute. Finally, 3 mL of the hexane phase containing the diffused monomers was carefully collected using a pipette and analyzed by UV-vis spectrometer. The illustration of UV-vis diffusion experiment and the related results were shown in Figure S1.

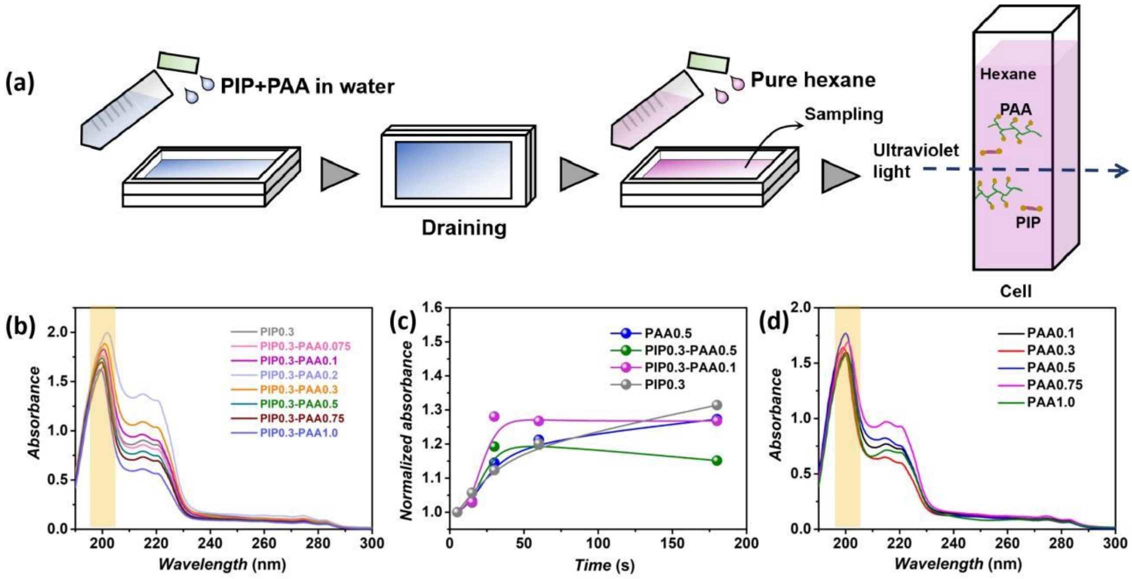

Figure S1. (a) Illustration of UV-vis measurement of diffusion experiment. (b) Absorbance of diffused amine in hexane with different PIP0.3-PAA. (c) Time-dependent absorbance variation of PIP0.3, PAA0.5, PIP0.3-PAA0.1 and PIP0.3-PAA0.5 in hexane solution and (d) Absorbance of diffused amine in hexane with different bare PAA concentrations over a period of 1 min.

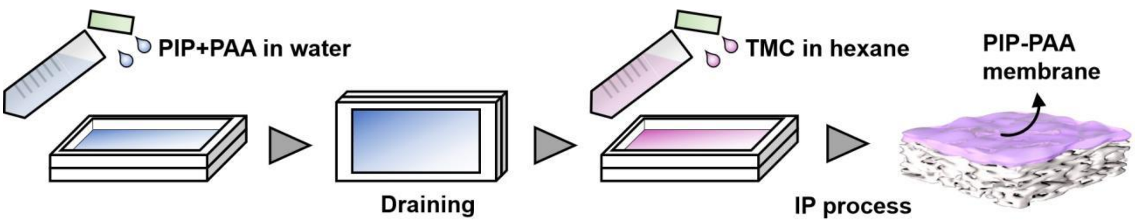

Figure S2. PIP-PAA membrane preparation process.

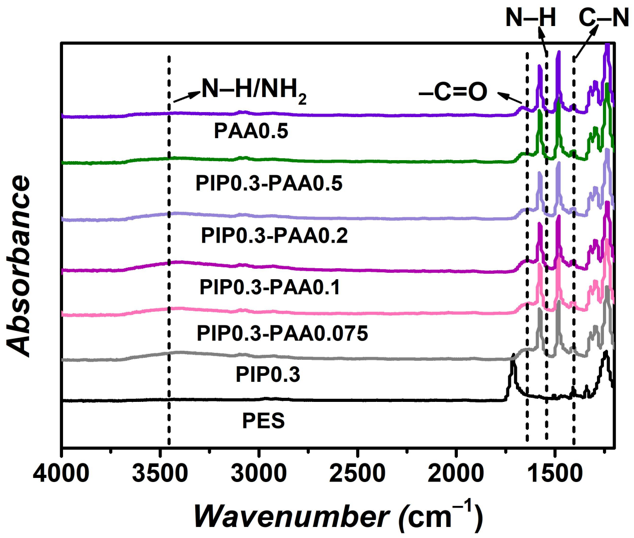

Figure S3. FTIR spectra of different membranes.

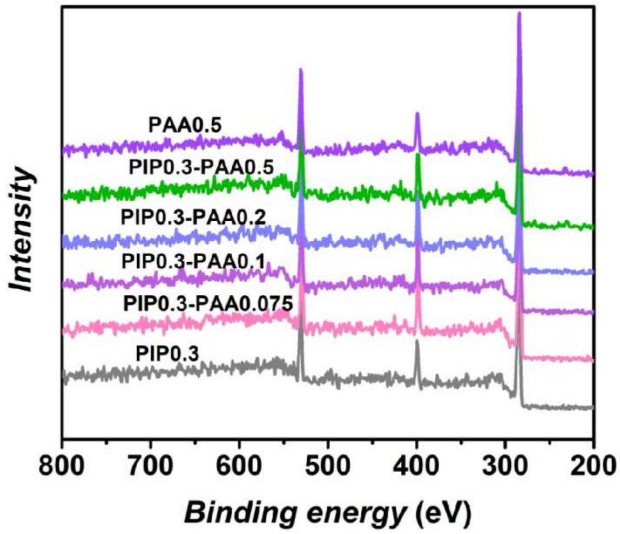

Figure S4. XPS survey spectra of different membranes.

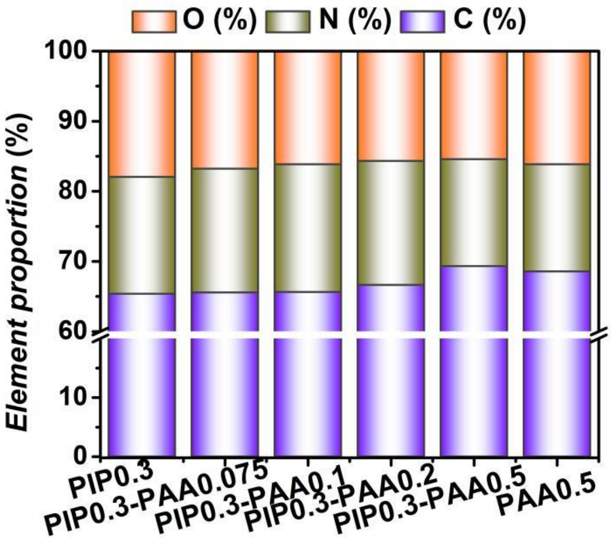

Figure S5. Surface chemical composition of different membranes obtained by XPS.

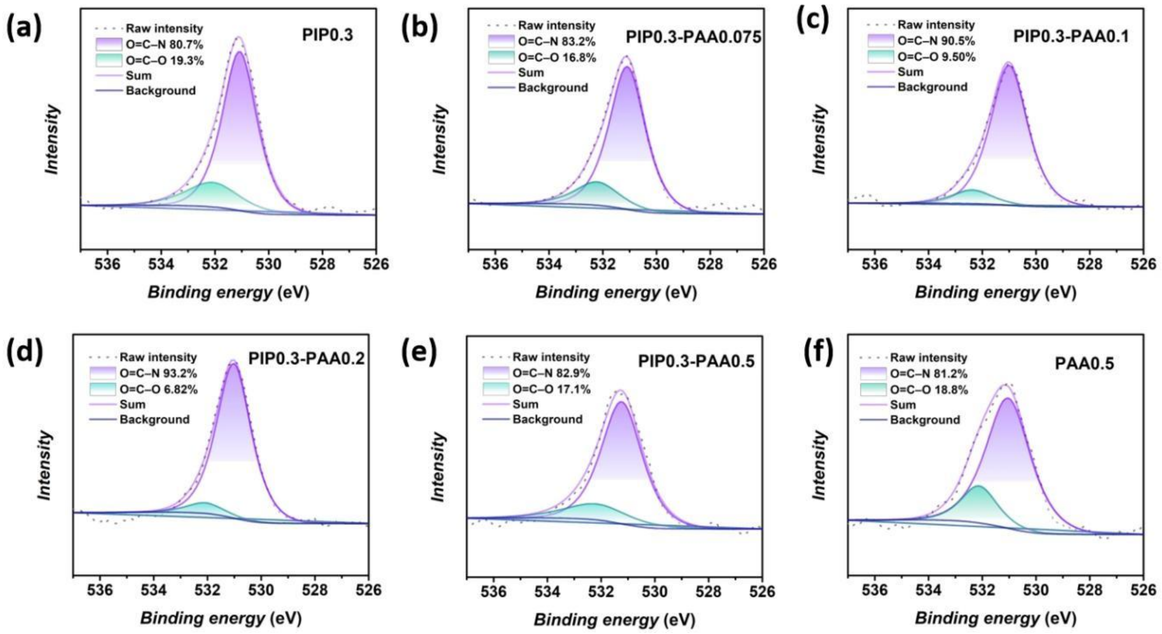

Figure S6. XPS O1s spectra of different membranes.

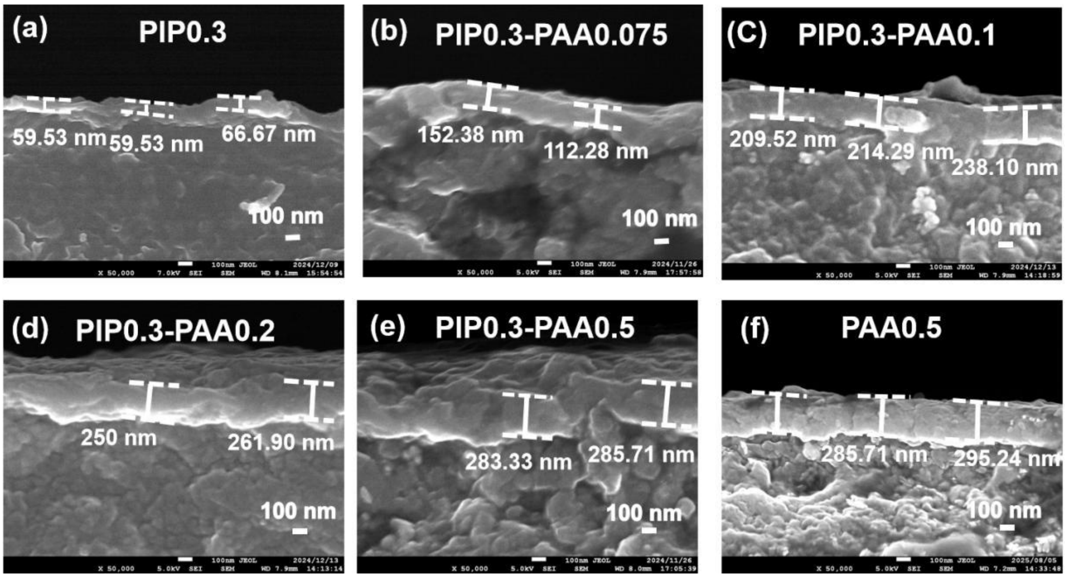

Figure. S7. Cross-sectional morphology of the PIP0.3, PIP0.3-PAA0.075, PIP0.3-PAA0.1, PIP0.3-PAA0.2, PIP0.3-PAA0.5, and PAA0.5 membranes.

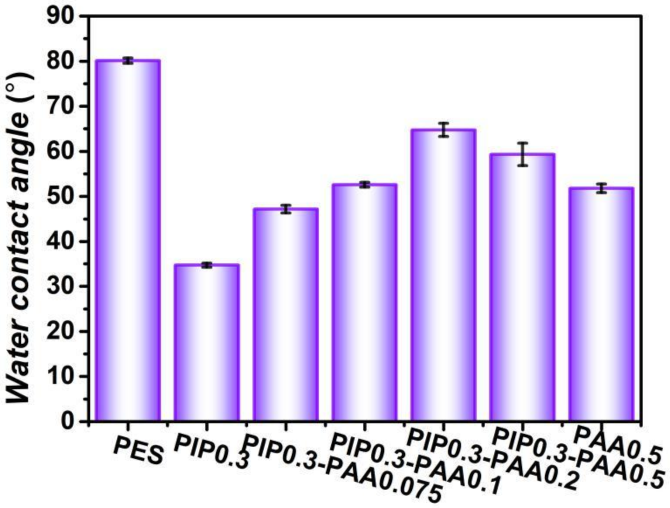

Figure S8. Water contact angle of different membranes. The data are shown as mean ± SD, n=3.

The PIP0.3 membrane exhibited the lowest WCA value of 34°, which may be due to the presence of a large number of carboxyl groups on the membrane surface. Importantly, it was observed that as the incorporation of PAA monomer increase, the WCA values of the PIP-PAA membranes gradually rose, despite the formation of nano-wrinkled structures. This increase in WCA values can be partially attributed to the higher crosslinking degree, which reduced the number of carboxyl groups. However, as for PIP0.3-PAA0.5 membrane, the surface hydrophilicity increased compared to PIP0.3-PAA0.2, which was primarily due to the abundant amine groups on the membrane surface. Additionally, the pure PAA membrane exhibited increased hydrophilicity with a smooth structure. The enhanced hydrophilicity is due to the significant presence of amine groups on the membrane surface.

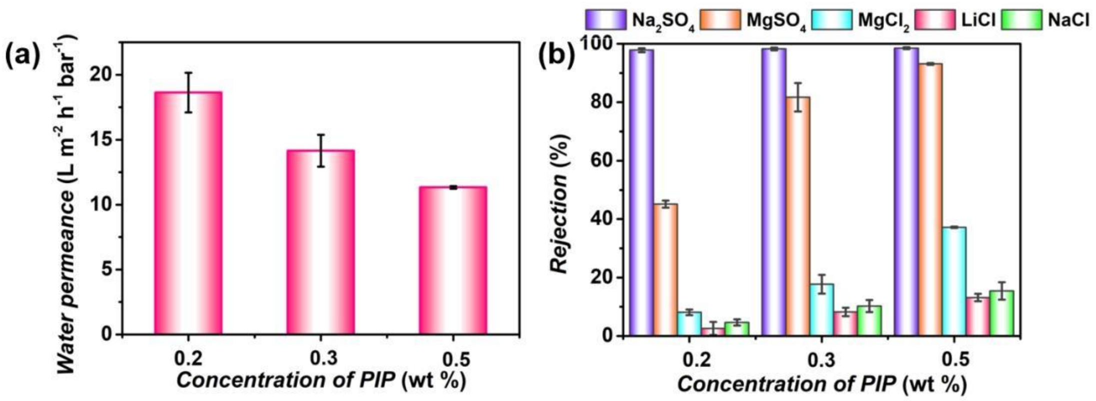

Figure S9. (a) Water permeance, (b) Na_2SO_4, $MgSO_4$, $MgCl_2$, LiCl, NaCl rejections of prepared PIP0.2, PIP0.3, PIP0.5 membrane. (Feed solution: each 2000 ppm single salt solution). The data are shown as mean ± SD, n=3.

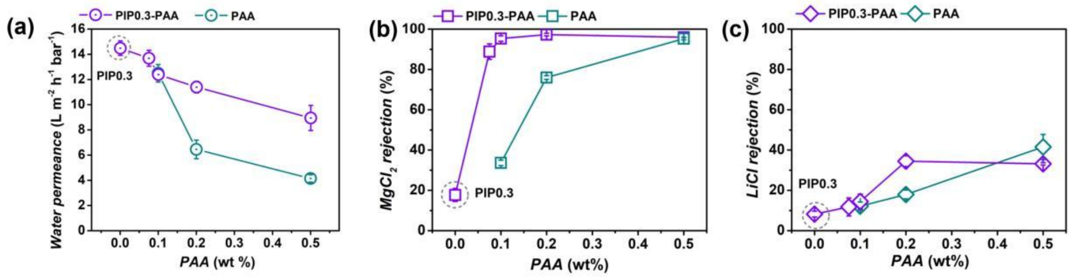

Figure S10. (a) Water permeance, (b) $MgCl_2$ rejections, (c) LiCl rejections of prepared PIP0.3, PIP0.3-PAA (0.075─0.5 wt%), and PAA (0.1─0.5 wt%) membranes, respectively. (Feed solution: 2000 ppm $MgCl_2$ or 2000 ppm LiCl). The data are shown as mean $\pm$ SD, n=3.

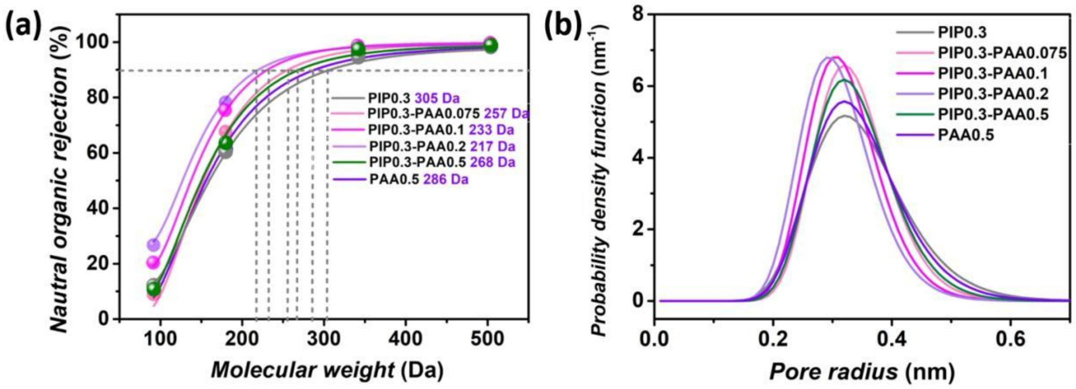

Figure S11. (a) MWCO curves and (b) pore size distribution of different membranes.

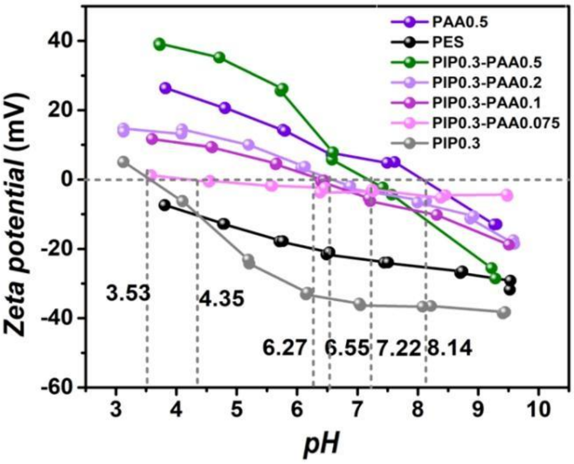

Figure S12. Surface zeta potential of PES support and different NF membranes under different pH condition.

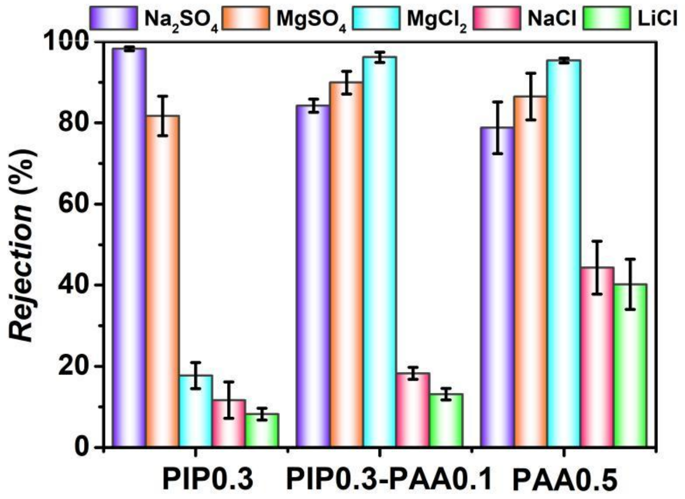

Figure S13. Rejections of the PIP0.3, PIP0.3-PAA0.1, and PAA0.5 membranes with each 2000 ppm single salt feed solution ($MgCl_2$, LiCl, $MgSO_4$, Na_2SO_4, NaCl). The data are shown as mean ± SD, n=3.

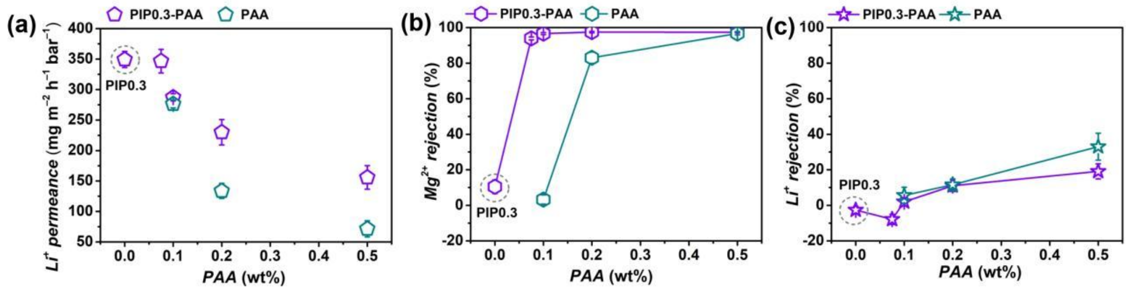

Figure S14. (a) Li^+ permeance, rejections for (b) Mg^{2+} and (c) Li^+ using PIP0.3, PIP0.3-PAA (0.075–0.5 wt%), and PAA (0.1–0.5 wt%) membranes. Feed solution: 2000 ppm $MgCl_2$ and LiCl mixture solution with a Mg^{2+}/Li^+ mass ratio of 20. The data are shown as mean ± SD, n=3.

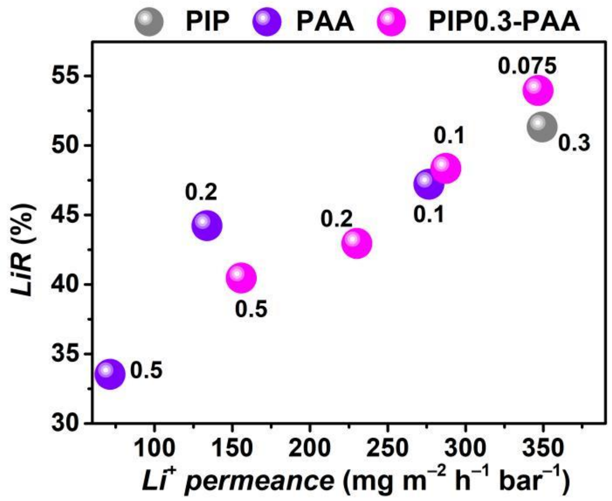

Figure S15. Plot of *LiR* versus Li^+ permeance of prepared PIP0.3, PIP0.3-PAA (0.075–0.5 wt%), and PAA (0.1–0.5 wt%) membranes. Feed solution: 2000 ppm $MgCl_2$ and LiCl mixture solution with a Mg^{2+}/Li^+ mass ratio of 20. The *LiR* calculated here assumes that the rejections of Li^+ and Mg^{2+} are independent of water recovery, with the membrane's water recovery set at 50%. The data are shown as mean values, n=3, with error bars excluded from the plots for clarity.

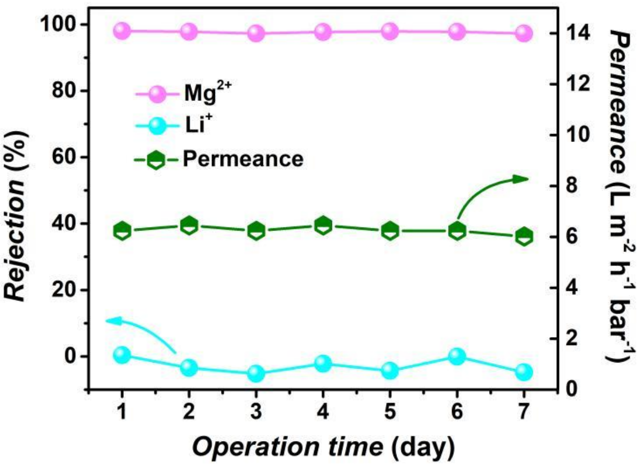

Figure S16. 7-day continuous separation test by PIP0.3-PAA0.1 membrane (Feed solution: 2000 ppm mixed $MgCl_2$ and LiCl solution at a Mg^{2+}/Li^{+} mass ratio of 20).

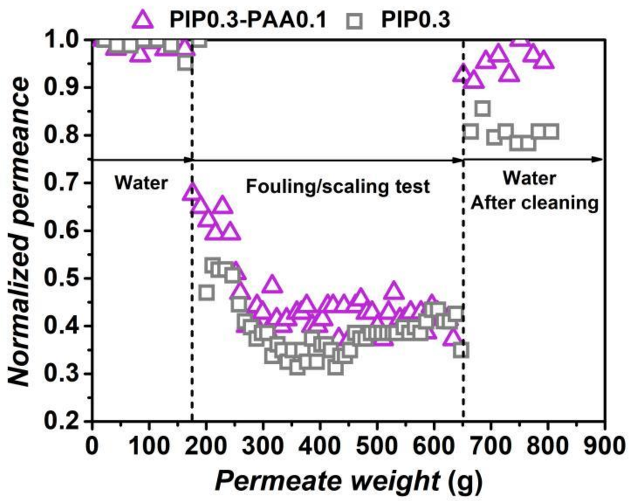

Figure S17. Permeance decline curves of the PIP0.3 and PIP0.3-PAA0.1 membranes during the inorganic fouling/scaling test (feed solution containing 300 mg L^{-1} Na_2SiO_3, 500 mg L^{-1} $CaSO_4$, 500 mg L^{-1} $MgCl_2$, and 500 mg L^{-1} LiCl; pH 6.6, 30 °C, 5 bar).

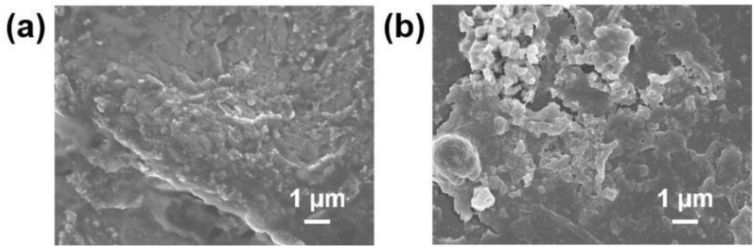

Figure S18. Surface SEM morphologies of the (a) PIP0.3 and (b) PIP0.3-PAA0.1 membranes after inorganic fouling/scaling tests.

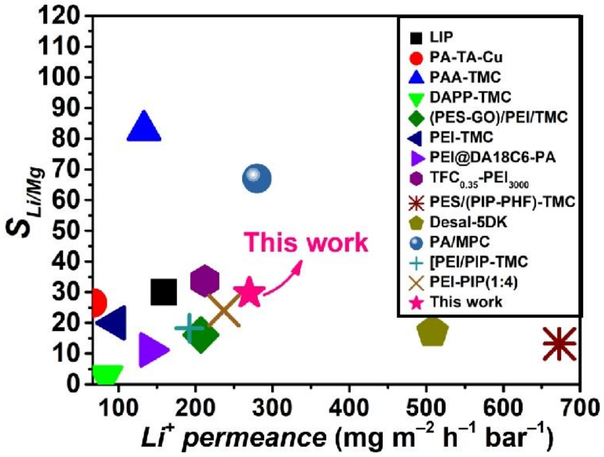

Figure S19. Separation performance compared with those reported in other literature (for more details, see Table S2 in the Supplementary Information).

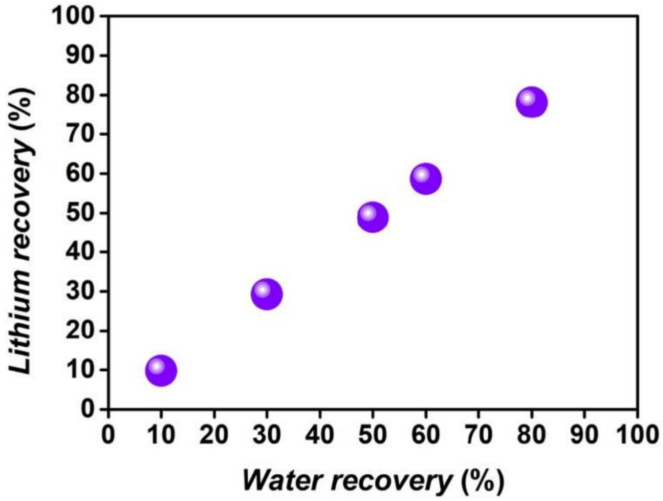

Figure S20. Lithium recovery dependence on water recovery in the two-stage NF process.

Table S1. Elemental composition on the surface of NF membranes.

Membrane	C (%)	N (%)	O (%)	N/O
PIP0.3	65.4	16.6	18.0	0.922
PIP0.3-PAA0.075	65.6	17.6	16.8	1.05
PIP0.3-PAA0.1	65.6	18.2	16.2	1.12
PIP0.3-PAA0.2	66.6	17.7	15.7	1.13
PIP0.3-PAA0.5	69.3	15.3	15.4	0.994
PAA0.5	68.6	15.3	16.1	0.950

Table S2. Comparison with other literature reports.

Membranes	Water permeance (L m^{-2} h^{-1} bar^{-1})	Rejection to $MgCl_2$ (%)	Rejection to LiCl (%)	$S_{Li/Mg}$	Rejection to Li^+ (%)	Li^+ Permeance (mg m^{-2} h^{-1} bar^{-1})	Ref.
LIP	10.2	95.9	21.0	29.9	34.0	159	[1]
PA-TA-Cu	4.87	95.9	17.1	26.5	42.0	66.3	[2]
PAA-TMC	7.39	97.3	14.6	82.8	23.3	133	[3]
DAPP-TMC	2.53	70.4	21.8	2.6	-40.7	83.6	[4]
(PES-GO)/PEI/TMC	11.2	~94	~29.5	16.12	20.9	207	[5]
PEI-TMC	5.02	94.8	34.6	20.0	19.0	95.5	[6]
PEI@DA18C6-PA	10.4	96.3	43.4	11.2	~42.0	141	[7]
$TFC_{0.35}$-PEI_{3000}	9.60	97.0	60.2	33.6	5.90	212	[8]
PES/(PIP-PHF)-TMC	6.70	89.9	16.3	13.2	25.0	673	[9]
Desal-5DK	8.20	95.1	~10	17.1	84.2	508	[10]
PA/MPC	8.50	97.1	5.26	67.0	-40.0	280	[11]
PEI/PIP-TMC	10.6	~95.0	~30.0	18.26	~23.0	192	[12]
PEI-PIP (1:4)	15.0	98.5	~20.0	24	32.8	237	[13]
PIP0.3-PAA 0.1	12.1	94.7	12.6	29.7	4.60	270	This work

Table S3. Cation concentration for each stage of the two-stage process.

Two-stage NF process	Mg^{2+}(ppm)	Ca^{2+}(ppm)	Na^{+}(ppm)	K^{+}(ppm)	Li^{+}(ppm)
Feed	1200	45.1	241	241	30.0
1st NF	80.2	8.54	317	366	42.6
2nd NF	2.15	0.23	205	226	29.3

Table S4. Amine monomer concentrations used for preparation of different NF membranes.

Type	Membrane	PIP concentration	PAA concentration
PIP	PIP0.2	0.2 wt%	0
	PIP0.3	0.3 wt%	0
	PIP0.5	0.5 wt%	0
PIP0.3-PAA	PIP0.3-PAA0.075	0.3 wt%	0.075 wt%
	PIP0.3-PAA0.1	0.3 wt%	0.1 wt%
	PIP0.3-PAA0.2	0.3 wt%	0.2 wt%
	PIP0.3-PAA0.5	0.3 wt%	0.5 wt%
PAA	PAA0.1	0	0.1 wt%
	PAA0.2	0	0.2 wt%
	PAA0.5	0	0.5 wt%

References

[1] S. Duan, S. Jiang, Z. Li, P. Zhang, K. Guan, P. Xu, H. Matsuyama, *Desalination* **2025**, 597, 118393, https://doi.org/10.1016/j.desal.2024.118393.

[2] S. Fang, K. Guan, S. Zhou, Q. Song, Y. Shi, W. Fu, Z. Li, P. Xu, M. Hu, Z. Mai, P. Zhang, H. Matsuyama, *Sep. Purif. Technol*. **2024,** 581, 117577, https://doi.org/10.1016/j.desal.2024.117577.

[3] P Xu, R. R. Gonzales, J. Hong, K. Guan, Y-H. Chiao, Z. Mai, Z. Li , S. Rajabzadeh, H. Matsuyama, *J. Membr. Sci.* **2023,** 668, 121251, https://doi.org/10.1016/j.memsci.2022.121251.

[4] X. Li, C. Zhang, S. Zhang, J. Li, B. He, Z. Cui, *Desalination* **2015,** 369, 26-36. https://doi.org/10.1016/j.desal.2015.04.027.

[5] P. Xu, J. Hong, X. Qian, Z. Xu, H. Xia, Q. Q. Ni, *Desalination* **2020,** 488, 114522, https://doi.org/10.1016/j.desal.2020.114522.

[6] P. Xu, W. Wang, X.M. Qian, H.B. Wang, C.S. Guo, N. Li, Z.W. Xu, K.Y. Teng, Z. Wang, *Desalination* **2019,** 449, 57-68, https://doi.org/10.1016/j.desal.2018.10.019.

[7] Z. Zha, T. Li, I. Hussei, Y. Wang, S. Zhao, *J Membrane Sci*, **2024,** 695, 122484, https://doi.org/10.1016/j.memsci.2024.122484.

[8] L. Li, G. Zhu, Y. Tong, K. Ding, Z. Wang, C. Meng, C. Gao. *J. Water Process Eng.* **2023,** 54, 103894, https://doi.org/10.1016/j.jwpe.2023.103894.

[9] Q. Shen, S. Xu, Z. Xu, H. Zhang, Z. Dong, *J. Appl. Polym. Sci.* **2019,** 136(41), 48029, https://doi.org/10.1002/app.48029.

[10] M.A. Ashraf, X. Li, J. Wang, S. Guo, B.-H. Xu, *Sep. Purif. Technol.* **2020,** 247, 116965, https://doi.org/10.1016/j.seppur.2020.116965.

[11] P. Xu, S. Duan, Z. Li, M. Hu, P. Zhang, L. Dai, Z. Mai, K. Guan, and H. Matsuyama, *Adv. Funct. Mater.* **2024,** 35(10), 2416458. https://doi.org/10.1002/adfm.202416458.

[12] C. Guo, Y. Qian, P. Liu, Q. Zhang, X. Zeng, Z. Xu, S. Zhang, N. Li, X. Qian, and F. Yu, *ACS Appl. Mater. Interfaces* **2023**, 15(3), 4814-4825, https://doi.org/10.1021/acsami.2c19956.

[13] T. Qi, X. Chen, T. Lu, D. Jin, R. Xu, J. Zhong, *J. Membr. Sci.* **2024,** 706, 122930, https://doi.org/https://doi.org/10.1016/j.memsci.2024.122930.
